# Book Reviews

**Published:** 1894-05

**Authors:** 


### Book Reviews.

---

Treatment of the Diseases of the Stomach and Intestines. By A. Mathieu, Physician to the Paris Hospitals. Medical Practitioners' Library. Octavo, 285 pages. Parchment muslin, price, $2.50; flexible leather, gilt top, price, $3.25. New York: William Wood & Company. 1894.

With the methods and habits of life incident to the present civilization, there has been an enormous increase in diseases of the digestive tract. Keeping pace with the progress of events, a number of physicians have paid special attention to the studies of these diseases and have distinguished themselves in their successful management. The author of this treatise has had exceptional experience in this branch of medicine, and his work has been well received on the other side of the Atlantic. In this country there is a wide field for the dissemination of such literature, and we hope that this excellent treatise will be carefully studied by teachers and practitioners of medicine.

The author commences with diagnostic technique, to the consideration of which Part I., consisting of thirty-seven pages, is entirely devoted. It is a concise and intelligent exposition of this branch of the subject. General considerations on diet, comprising Part II., present much material for the careful consideration of every family doctor. In Part III. the author takes up the principal clinical forms of dyspepsia, and in nine chapters gives a patient elucidation of the symptoms and treatment of the common forms of gastro-intestinal diseases. Not the least interesting portion of this branch of the subject is the chapter on gastro-intestinal antisepsis. The fermentation of food after ingestion is so common, and decomposing food is so liable to give rise to poisoning or to infection, that we hail with special pleasure any promise of prevention or relief that may be offered. This author affirms that intestinal antisepsis, though doubted by many, is a palpable fact capable of actual demonstration.

The final section of the book is devoted to the consideration of the diseases of the stomach and intestine, to which Part IV., consisting of nine chapters, is given up. In Chapter VI., typhlitis, perityphlitis or appendicitis is considered in a very brief and, to our mind, unsatisfactory manner. While this disease has, of late, fallen under the domain of surgery so far as treatment is concerned, yet the general practitioner of medicine is usually first called in to these cases, hence should be armed with quite as much knowledge pertaining to their diagnosis, pathology and treatment as the surgeon is supposed to possess. The surgical technique of these cases is usually very simple, whereas the judgment that decides in a particular case just when to operate needs to be of the most trained and acute order, and this must ever be the province of the family doctor.

We commend this treatise to the careful study of every physician who deserves the name.

Text-Book of Normal Histology: Including an account of the Development of the Tissues and of the Organs. By George A. Piersol, M. D., Professor of Anatomy in the University of Pennsylvania. With 409 illustrations, of which 358 are from original drawings by the author. Philadelphia: J. B. Lippincott Company. 1893.

Among the many excellent text-books on general histology this effort of Professor Piersol's must be considered the best. It is a pleasure to peruse a book bristling with original observations and descriptions and, above all, original drawings. It seems to assure one that, although the classical illustrations are still classical, other observers have seen the same conditions and verified them.

Professor Piersol has been very methodical in the arrangement of his chapters, beginning with the cell, describing its structure, growth and peculiarities. The tissues, epithelial, connective, muscular and nervous, are described in the following chapters. The epithelial tissues are very properly classified as: (1) squamous; (2) columnar; (3) modified, as (*a*) ciliated; (*b*) goblet; (*c*) pigmented; and (4) specialized (*a*) glandular epithelium; (*b*) neuro-epithelium. This classification of the epithelia is free from ambiguity and cannot fail to clear up in the minds of students much of the darkness surrounding this subject. The chapters on the nervous elements are thorough, and full credit is given to American investigators, especially Schmidt, whose discovery of the segments of the medulla in nerve fibers was followed by Lantermann's independent discovery of the same phenomena, but who receives little or no credit by the continental writers. Many of the illustrations on the histology of the brain are made from sections stained with the Golgi method, and show the beautiful results obtained by this method in staining the ganglion cells and neuroglia cells. The pineal gland is regarded as a rudimentary sense organ. Whether this opinion will hold true is questionable, and, perhaps, the best way of dealing with this organ, for the present at least, would be to say, "function unknown." The histology of the eye is admirably given, as are also the descriptions of the other organs of sense.

An appendix follows, giving the most useful histological methods for hardening, imbedding, staining and finishing, including the best of the newer methods. We fail, however, to find the Pal method mentioned among these.

Taken as a whole, no histology as yet published contains so much useful information so skilfully treated and arranged as does this American work. Both author and publishers are to be congratulated. W. C. K.

---

THE INTERNATIONAL MEDICAL ANNUAL AND PRACTITIONER'S INDEX. A Work of Reference for Medical Practitioners. Twelfth year. Pp. 704. Price, $2.75. New York: E. B. Treat, 5 Cooper Union; Chicago: 199 Clark street. 1894.

The Annual has become of such universal use, that the readers of the JOURNAL need no introduction to its pages, no words of praise for its excellence and its breadth of knowledge on medical topics. With each succeeding year the reviewer notices an increase in the number of editors and contributors, in photographic plates, wood cuts, and in the number of pages, and yet we notice the familiar $2.75 remaining the same. If the price of the annual had kept pace with its advances in quality and quantity, the Annual of 1894 would be a more expensive work.

Synopsis of contents and special contributors for 1894 are as follows: Part 1. The Dictionary of New Remedies, containing a complete report of all new therapeutic agents introduced during the year, with clinical indications for their use, and a general review of therapeutics, by H. A. Hare, M. D., Philadelphia, professor of therapeutics in the Jefferson Medical College.

Part 2. The Dictionary of New Treatment. A complete index of diseases, showing the latest therapeutic recommendations (medical and surgical), enriched with numerous illustrations and colored plates wherever useful for the elucidation of the text.

Part 3. Sanitation. A record of the year's work in sanitary science, with particulars of recent improvements in sanitary appliances, by Joseph Priestley, B. A., M. D., D. P. H., medical officer of health and public analyst for Leicester, England.—Progress of Pharmacy, by F. F. W. Koch, New York, Editor of *Notes on New Remedies.*—New Inventions and Appliances, by Irving S. Haynes, M. D., demonstrator of anatomy, Medical Department University of the City of New York.—Books of the Year. A list of the chief medical works published during the year, with particulars and prices.

Especially to be commended are the plates accompanying Dr. Shaw's article on insanity. Of late years there has grown a tendency to diagnosticate the different forms of insanity by the facial expression. Those who are in a position to know realize with what difficulty it is to procure photographs of the excited states, and in studying the plates in Dr. Shaw's article one is well repaid for the cost of the work. The article on diseases of the ear is well written and handsomely illustrated.

To those who are acquainted with the former editions this one will be heartily welcomed, and to those unacquainted with the work the reviewer recommends it as one of the best and cheapest of medical publications. W. C. K.

---

A Primer of Psychology and Mental Disease. By C. B. Burr, M. D., Medical Superintendent Eastern Michigan Asylum ; member of the American Medico-Psychological Association, the State Medical Society, the Pontiac Medical Society ; corresponding member of the Detroit Medical and Library Association. Detroit : George S. Davis, Publisher.

In this little book of about one hundred pages Dr. Burr has sought to simplify, for attendants and nurses upon the insane, the abstruse subjects of psychology and mental disease.

The book is divided into three parts—namely, psychology, insanity, and management of cases of insanity. Part I., dealing with the faculties of the mind and with the divisions of the intellect—namely, sensation, perception, memory, ideation, reason, is, from the nature of the subject, the most difficult to treat clearly, and to say that a measure of success has been attained is commendation. The description of the divisions of the intellect is assisted by illustrations which are of considerable advantage, but we think some of the examples, notably those under the head of reasoning, might have been made more simple.

In Part II. the forms of insanity, and the characteristics of each, are clearly and admirably treated, and in a way which makes the book well adapted to class-room instruction. Attention is called to the insanities of the different physiological epochs of life,—pubescent, adolescent, climacteric,—and the degree of involvement of perception, memory and judgment, in the different forms of mental disease are scientifically and systematically outlined.

Part III. is full of valuable suggestions, derived from years of experience in the management of the insane, and, well studied, cannot help proving beneficial to nurses and to patients under their care.

We believe the little volume will be of great assistance to those whose duty it is to teach classes in training-schools, and also to nurses in their daily work. We do not think the preface any addition to the book. To the beginner we fear it must be somewhat depressing, for he must needs study of "concepts" further on, in order to read it intelligently. For him, the preface should be placed at the end of the book rather than at the beginning.

The work of the publisher is good and in keeping with the literary and scientific character of the book. A. W. H.

---

How to Use the Forceps, with an Introductory Account of the Female Pelvis and of the Mechanism of Delivery. By Henry G. Landis, A. M., M. D., Professor of Obstetrics and Diseases of Women and Children in Starling Medical College, Columbus, O. Revised and enlarged by Charles H. Bushong, M. D., Assistant Gynecologist and Pathologist to Dewitt Dispensary, New York. Small 8vo, pp. 203; illustrated. Price, $1.75. New York: E. B. Treat, Publisher, 5 Cooper Union. 1894.

It is important to arm the student of obstetrics, as well as the young practitioner of the obstetric art, with ample instructions on the subject of the forceps. It is an instrument in more general use than any other in the lying-in chamber, and it is one in which an astonishing amount of ignorance prevails regarding the methods of its application. It would seem as though a question so entirely governed by mechanical laws ought to be of universal and uniform application. It is to be feared that obstetric teachers have not always been clear enough in their instruction regarding the use of the forceps, hence students, in many instances, have gone out from the schools with indifferent, imperfect or false notions concerning the proper application of this humane and important aid to delivery.

The book before us has been in the hands of the profession since 1880, hence is no stranger seeking introduction through formal and technical review. While it is probable that the distinguished author, were he living, would modify some of the statements made in his valuable book and would elaborate others, it must ever remain to his credit that he was the first American author to present a monograph on the subject that the title of his book bears. It is a work full of useful information, and suggests the propriety of greater elaboration both in text and in illustration. There are some very good outlined drawings illustrative of the passage of the fetal head through the pelvis, but much more might be done to illustrate the text through the aid of the photograph and the heliotype. The editor has made a few additions to the work, in an attempt to bring it forward to the present period, but there remains yet much to be done to make it all that could be desired in this respect. Notwithstanding its imperfections, it is the best book of the kind and should be possessed by all obstetricians.

---

HOLDEN'S ANATOMY. A Manual of the Dissections of the Human Body. By JOHN LANGTON, F. R. C. S., Surgeon to, and Lecturer on, Anatomy at St. Bartholomew's Hospital. Carefully revised by A. Hewson, M. D., Demonstrator of Anatomy, Jefferson Medical College; Chief of Surgical Clinic, Jefferson Hospital; Member Association American Anatomists, etc. Sixth edition; 311 illustrations. Small 8vo, pp. xx.—803. Price, $3.00. Philadelphia: P. Blakiston, Son & Co., 1012 Walnut street. 1894.

The sixth edition of this work, now before us, is an index of its popularity. The first edition was issued in 1851, and while six editions of an ordinary medical work in forty-three years are not many, yet for an anatomy it speaks volumes. The human body is not changeable as to its form or functions, yet new anatomical features are occasionally coming to light, and methods of teaching are constantly changing. Hence, an occasional new edition of a treatise on anatomy becomes necessary.

The special purpose of the author in issuing this manual was to place in the hands of the student a suitable working book for the dissecting-room. In it he has endeavored to direct attention to the prominent facts of anatomy and to teach the ground-work of the science; to trace the connection and to point out the relative situation of parts without perplexing the student with minute descriptions. It is in no sense intended to supplant the more complete anatomical works or to fill the place for which they are intended. It is an excellent working book for the dissecting-room and is not excelled by any extant with which we are familiar.

The editor of the sixth edition has taken great pains to bring the work abreast of the present time, and has put an enormous amount of labor into its revision, for which he deserves great credit.

---

TRANSACTIONS OF THE AMERICAN ASSOCIATION OF OBSTETRICIANS AND GYNECOLOGISTS. Volume VI. for the year 1893. Edited by WILLIAM WARREN POTTER, M. D., Secretary. Philadelphia: William J. Dornan. 1894.

This volume, like its predecessors, is full of interest to the obstetrician, gynecologist, abdominal and pelvic surgeon. The president's annual address, entitled The Present Position of Pelvic

Surgery, by L. S. McMurtry, M. D., Louisville, is an interesting and able exposition of the subject, and deserves to be carefully read by every interested physician. One of the attractive features of the transactions of this association, and the present volume is no exception to the rule, is the great strength of the discussions. These serve to make the text of the papers stand out in bold relief, and are full of instruction, as all just criticisms are and ever must be. As illustrating this point, we may instance the discussion on the papers of Drs. Longyear and Reed, relating to the closure and management of the abdominal incision. The text of both papers is strong and the discussions are searching.

The volume is well illustrated, though there is room for further elaboration in this department. It contains a memorial of Dr. George Jackson Fisher, accompanied by a full-page lithographic portrait of this remarkable man.

Those who desire to obtain this volume should make early application for it, as the edition is limited.

---

The Year-Book of Treatment for 1894. A Comprehensive and Critical Review for Practitioners of Medicine and Surgery. In a series of twenty-four chapters, by eminent specialists. In one 12mo volume of 497 pages. Cloth, $1.50. Philadelphia: Lea Brothers & Co. 1894.

This book has appeared with promptitude for the last ten years, so that now it has become firmly established as a reference book of treatment, which is, as its title indicates, a critical review of that subject in its broadest sense. It is a valuable aid to every physician who would keep abreast of the progress making in knowledge relating to the management of disease.

It consists of twenty-four chapters, each contributed by a writer eminent in his assigned subject. These are grouped mainly from English sources, but their work is of such a nature as to embrace the literature of the world. Some of the articles are written in much detail, but references to the original source are given in all instances, making it convenient to pursue extended research whenever desired. The book contains a selected list of new books, new editions and translations, and the volume is closed by an index of authors quoted, as well as an index of subjects. Those who have possessed themselves of the previous volumes will make haste to obtain this, while all will find it an exceedingly useful book of reference.

A Practical Treatise on the Diseases of the Hair and Scalp. By George Thomas Jackson, M. D., Professor of Dermatology, Woman's Medical College, New York Infirmary; Chief of Clinic and Instructor in Dermatology, College of Physicians and Surgeons; Consulting Dermatologist, Presbyterian Hospital; Visiting Dermatologist, Randall's Island Hospital; Member of the American Dermatological Association, etc. New revised and enlarged edition. Small 8vo, pp. 414. New York: E. B. Treat, 5 Cooper Union. 1893. Price, $2.75.

The proper care of the hair is a very important part of the knowledge that should be possessed by the dermatologist, and to a very large extent by the general practitioner of medicine. The author of this book is well known to the profession as a competent teacher as well as practiser of dermatology. When the first edition of his work was issued, it was about the first scientific treatise known on the subject. Since it was issued in March, 1887, knowledge on the subject has increased in a vast degree, rendering a new edition absolutely necessary to keep up with the advances making in this department of medicine.

The author states that every page of the old edition has been revised and corrected; that new articles upon folliculitis decalvans, lepothrix and aplasia pilorum propria, and many new sections to the old chapters, have been added; that the bibliography has been brought down to January, 1893, and nine new illustrations have been inserted in the text.

This work deserves to be found on the book-shelves of every progressive physician.

---

A Practical Treatise on Nervous Exhaustion (Neurasthenia): Its Symptoms, Nature, Sequences, Treatment. By George M. Beard, A. M., M. D., Fellow of the New York Academy of Medicine, of the New York Academy of Sciences; Vice-President of the American Academy of Medicine; Member of the American Neurological Association, of the American Medical Association, the New York Neurological Society, etc. Edited, with notes and additions, by A. D. Rockwell. A. M., M. D., Professor of Electro-Therapeutics in the New York Post-Graduate Medical School and Hospital; Fellow of the New York Academy; Member of the American Neurological Association, of the New York Neurological Society, etc. Third edition, enlarged. Small 8vo, pp. 262. Price, $2.75. New York: E. B. Treat, 5 Cooper Union. 1894.

After five years, a new edition of this instructive and readable book makes its appearance. When Beard first called attention to neurasthenia in a paper published in the *Boston Medical and Surgical Journal*, April, 1869, it attracted absolutely no attention whatever. Now, after twenty-five years, so-called nervous prostration has become almost a fashionable disease. At all events, a certain group of symptoms commonly so termed, but professionally named as neurasthenia, is met by almost every physician in his daily practice.

The author is especially clear and succinct in dealing with the diagnosis of neurasthenia, while the editor has succeeded in bringing the work well down to the present through his own contributions and additions to the subject. It is an excellent treatise for the general practitioner to possess, and the specialist cannot afford to be without it.

---

THE MICROSCOPE AND MICROSCOPICAL METHODS. By SIMON HENRY GAGE, Associate Professor of Anatomy, Histology and Embryology in Cornell University, Ithaca, N. Y. Fifth edition, rewritten, greatly enlarged and illustrated by 103 figures in the text. Part I. of The Microscope and Histology. Ithaca, N. Y.: Comstock Publishing Co. 1894.

The fourth edition of Professor Gage's Histology was reviewed at some length in the January (1892) number of the JOURNAL, and its many excellent qualities there set forth. The fifth edition has been in part rewritten and enlarged, so that it is nearly one-half larger than the preceding edition. This has been done to bring the subject up to the present state of the science and to incorporate a new chapter on photography and photo-micrography. The importance of photography to a microscopist is becoming every day more apparent, and the able treatment of this chapter in Professor Gage's book will help materially to bring the two sciences in closer relation.

On the whole there is, perhaps, no book that will ground the beginner so thoroughly in microscopy as this work of Professor Gage. W. C. K.

---

ANTISEPTIC THERAPEUTICS. By DR. E. L. TROUESSANT, Paris, France. Translated by E. P. Hurd, M. D. In two volumes. Physicians' Leisure Library. Price, paper, 25 cents each; cloth, 50 cents each. Detroit, Mich.: George S. Davis. 1893.

THE MODERN CLIMATIC TREATMENT OF INVALIDS WITH PULMONARY CONSUMPTION IN SOUTHERN CALIFORNIA. By P. C. REMONDINO, M. D. Member of the American Medical Association, American Public Health Association, etc., etc. Physicians' Leisure Library. Price, paper, 25 cents; cloth, 50 cents. Detroit, Mich.: George S. Davis. 1893.

I. Antiseptic therapeutics is forming a large part in the practice of medicine as well as surgery. Many of its conditions are as yet unsettled, but it is well to have occasional expositions noting progress on the subject that shall keep pace with the advances in bacteriology. These two volumes by Trouessant, forming a portion of the Physicians' Leisure Library, will amply repay perusal, and are a remarkably cheap edition of valuable literature.

II. The recent studies in tuberculosis, that have shown it to be a communicable disease, lends added interest to any literature that is published regarding its treatment. If it is communicable, which cannot be denied, it is preventable, and if preventable it is or ought to be curable. Climate has always played an important part in its cure, and California within the last few years has attracted much attention in this regard on account of its salubrious climate. The author has set forth its claims in a readable fashion.

---

### BOOKS RECEIVED.

Gonorrhea: Being the Translation of Blenorrhea of the Sexual Organs and Its Complications. By Dr. Ernest Finger, Docent at the University of Vienna. One volume, of 330 pages, octavo, illustrated by numerous wood engravings, and by seven chromo-lithographic plates. Third revised and enlarged edition. Bound in muslin, gold lettered, $3.00. New York: William Wood & Company. 1894.

The Johns Hopkins Hospital Reports. Volume IV., No. 1. Report on Typhoid Fever. Quarto, pp. 167. Baltimore: The Johns Hopkins Press. 1894.

Transactions of the American Dermatological Association at its seventeenth annual meeting, held at the Hotel Pfister, Milwaukee, Wis., on the 5th and 6th of September, 1893. Edited by George Thomas Jackson, M. D., Secretary. Octavo, paper, pp. 82. New York. 1894.

Clinical Diagnosis. By Albert Abrams, M. D., Heidelberg; Professor of Pathology, Cooper Medical College, San Francisco; Pathologist to the City and County Hospital, San Francisco, etc., etc. Third edition, revised and enlarged. Illustrated. Small 8vo, pp. 273. Price, $2.75. New York: E. B. Treat, 5 Cooper Union. 1894.

A Manual of Practical Obstetrics. By Edward P. Davis, A. M., M. D., Professor of Obstetrics and Diseases of Infancy in the Philadelphia Polyclinic; Clinical Professor of Pediatrics in the Woman's Medical College; Clinical Lecturer on Obstetrics and Gynecology in the Jefferson Medical College, etc., etc. Second edition, revised and enlarged. With 134 illustrations and sixteen full-page plates, several of which are colored. Small 8vo, pp. xii.—351. Price, $2.50. P. Blakiston, Son & Co., 1012 Walnut street. 1894.

Pain in its Neuro-Pathological, Diagnostic, Medico-Legal and Neuro-Therapeutic Relations. By J. Leonard Corning, A. M., M. D., Consultant in Nervous Diseases to St. Francis' Hospital, St. Mary's Hospital, the Hackensack Hospital, etc., etc. Illustrated. Small 8vo, pp. 328. Philadelphia: J. B. Lippincott Company. 1894.

A Manual of Nursing in Pelvic Surgery. By Lewis S. McMurtry, A. M., M. D., Professor of Gynecology in the Hospital College of Medicine, Louisville; Surgeon-in-Charge of the Jennie Cassady Infirmary for Women; Gynecologist to Sts. Mary and Elizabeth Hospital, etc. Duodecimo, pp. 92. Morton's Pocket Series, No. 3. Louisville: John P. Morton & Company. 1894.

Transactions of the Southern Surgical and Gynecological Association. Volume VI. Sixth session. Held at New Orleans, La., November 14, 15 and 16, 1893. Octavo, pp. xlvii.—392. Edited by W. E. B. Davis, M. D., Secretary. Published by the Association. Philadelphia: W. J. Dornan, Printer. 1894.

Essentials of Practice of Pharmacy, arranged in the form of Questions and Answers. Prepared especially for pharmaceutical students. Saunders' Question Compends, No. 18. Second edition, revised. By Lucius E. Sayre, Ph. G., Professor of Pharmacy and Materia Medica of the School of Pharmacy of the University of Kansas. Duodecimo, pp. ix.—200. Price, $1.00. Philadelphia: W. B. Saunders, 925 Walnut street. 1894.

Asepsis in der Gynäkologie und Geburtshülfe. Von Dr. M. Sänger, ausserordentlicher Professor an der Universität, Leipzig, und Dr. W. Odenthal, in Hanover, früher Assistenzarzt an Prof. Sänger's Heilanstalt. With two plates and forty-two illustrations in the text. Leipzig: C. G. Naumann. 1894.

